# Extracranial carotid plaque hemorrhage predicts ipsilateral stroke recurrence in patients with carotid atherosclerosis – a study based on high-resolution vessel wall imaging MRI

**DOI:** 10.1186/s12883-022-02758-3

**Published:** 2022-06-28

**Authors:** Fengli Che, Donghua Mi, Anxin Wang, Yi Ju, Binbin Sui, Xiaokun Geng, Xihai Zhao, Xingquan Zhao

**Affiliations:** 1 Department of Neurology, Beijing Tiantan Hospital, Capital Medical University, Beijing, China; 2 Department of Neurology, Beijing Luhe Hospital, Capital Medical University, Beijing, China; 3 Tiantan Neuroimaging Center for Excellence, Beijing Tiantan Hospital, Capital Medical University, Beijing, China; 4 Department of Neuroradiology, Beijing Tiantan Hospital, Capital Medical University, Beijing, China; 5 Department of Biomedical Engineering, Center for Biomedical Imaging Research, Tsinghua University, Beijing, China; 6 Research Unit of Artificial Intelligence in Cerebrovascular Disease, Chinese Academy of Medical Sciences, Beijing, China

**Keywords:** High-resolution vessel wall MRI, Carotid atherosclerotic plaque, Intraplaque hemorrhage, Recurrence of stroke

## Abstract

**Background:**

Intraplaque hemorrhage (IPH) is a hallmark of carotid plaque vulnerability. We aim to investigate the association between IPH and recurrent ipsilateral ischemic stroke.

**Methods:**

Patients with a recent stroke or transient ischemic attack (TIA) were prospectively recruited and underwent an ultrasonographic examination and carotid HR VWMRI on the side consistent with symptoms. Carotid plaque was defined as carotid intima-media-thickness (IMT) by ultrasound≥1.5 mm. IPH was determined that the ratio of the plaque signal intensity relative to that of adjacent muscle was > 1.5. All enrolled patients were clinically followed until an ipsilateral ischemic stroke, TIA, carotid endarterectomy (CEA)/carotid artery stenting (CAS), or death within 12 months. Univariate analysis was used to analyze the correlation between clinical characteristics and IPH. Kaplan-Meier survival analysis and a log-rank test were used to compare recurrence-free survival time between the IPH and non-IPH groups. Cox regression models evaluated IPH as the predictor of ipsilateral stroke recurrence.

**Results:**

A total of 171 patients (mean age, 60.13 ± 10.04 years; 118 males) were included in the final analysis. Thirty-two patients (18.7%) showed carotid IPH. During the follow-up, patients with carotid IPH suffered 60.9% (14 of 23) of recurrent ipsilateral strokes and 60.0% (3 of 5) TIA. Multivariate Cox regression analysis proved IPH as a strong predictor of ipsilateral stroke; the adjusted hazard ratio (HR) was 6.64 (95% confidence interval [CI], 2.84–15.54, *P* < 0.001). Meanwhile, Cox regression analysis also proved that IPH could predict recurrent ischemic events; the adjusted HR was 8.08 (95% CI, 3.65–17.91, *P* < 0.001).

**Conclusions:**

Carotid intraplaque hemorrhage is strongly associated with recurrent ischemic events and could predict recurrent ipsilateral stroke.

**Supplementary Information:**

The online version contains supplementary material available at 10.1186/s12883-022-02758-3.

## Introduction

Atherosclerotic lesions are the leading cause of ischemic stroke, especially in the Chinese population [1, 2]. China National Stroke Registry (CNSR) study has shown that atherosclerotic stroke accounts for 60% of the etiology of ischemic stroke [3]. Several clinical studies have confirmed that carotid atherosclerosis stenosis is significantly correlated with the onset of acute ischemic stroke and clinical prognosis [4]. Consequently, the measurement of carotid artery stenosis has been used in many clinical trials for the risk stratification of stroke patients in the past 20 years [5–7].

More studies have confirmed that the vulnerable plaque features of carotid atherosclerosis are the potential risk factors for most ischemic events and are significantly correlated with the recurrence of ischemic events [8–13]. Therefore, identifying the vulnerable characteristics of carotid artery plaques based on advanced medical imaging technology and exploring the imaging markers that can predict the risk of stroke recurrence have become research focuses in recent years.

However, there are still few studies on the incidence of extracranial carotid intraplaque hemorrhage (IPH) in acute atherosclerotic ischemic stroke patients and whether there is a significant relationship between IPH and the recurrence of ipsilateral stroke. Our study aims to find the correlation between IPH and ipsilateral stroke recurrence, intending to identify the high-risk factors of stroke recurrence and make individualized prevention strategies.

## Methods

### Study population

Our study was an observational study of single-center. Patients with a recent stroke or transient ischemic attack (TIA) were recruited in Beijing Tiantan Hospital from June 2011 to December 2014. All patients underwent an ultrasonographic examination and carotid high-resolution vessel wall MRI (HR VWMRI) scan on the side consistent with symptoms within 7 days after admission. According to the recent guidelines from AHA/ASA, patients received standardized medical therapy for secondary stroke prevention and were followed up for 12 ±1 months. All patients fulfilled the following criteria: (1) age ≥ 18 years old; (2) onset of symptoms within the last 14 days; (3) National Institutes of Health Stroke Scale score (NIHSS) ≤ 22 when enrolled; (4) carotid intima-media-thickness (IMT) by ultrasound≥1.5 mm. Exclusion criteria: (1) non-cerebral infarction disease including intracranial hemorrhage (intracranial parenchymal hemorrhage, subarachnoid hemorrhage, subdural hematoma or epidural hematoma), tumor, infection, etc.; (2) suspected cardiogenic embolism or previous history of congenital heart disease, rheumatic heart disease, atrial fibrillation, etc.; (3) suspected non-atherosclerotic vascular lesions ((i.e., vasculitis, arterial dissection, or Moyamoya disease); (4) undergoing emergent thrombolysis or interventional treatments; (5) previous history of carotid endarterectomy or carotid stenting; (6) suffering from severe hepatic or renal insufficiency; (7) life expectancy < 1 year; (8) contraindication to MRI examination (including claustrophobia, metal placement in the body, etc.); (9) Severe stenosis or occlusion of the ipsilateral intracranial carotid artery or middle cerebral artery confirmed by magnetic resonance artery (MRA). The Ethics Committee of Beijing Tiantan Hospital approved this study. All study subjects had signed informed consent.

### Clinical information assessment

We obtained the patients’ pre-existing conditions from themselves, their relatives, or caregivers. We recorded the clinical assessments for ischemic cerebrovascular events (stroke/TIA), cardiovascular risk factors, and medications during recruitment. We defined the present conditions following the definitions recommended by the related international guidelines: hypertension was determined by the diagnosis at discharge or a history of hypertension [14]; diabetes mellitus was defined by the diagnosis at discharge or a history of diabetes mellitus [15]; hyperlipidemia was defined as low-density lipoprotein (LDL) ≥1.7 mmol/L at admission or a history of hyperlipidemia or receiving lipid-lowering treatment or diagnosis at discharge [16]; coronary artery disease was defined as the previous history of angina pectoris or myocardial infarct [17]; smoking history was defined as continuous or cumulative smoking for more than 6 months in one’s life [18]; family history of stroke was defined as at least one of a patient’s first-grade relatives having a history of stroke [19]. The stroke etiology was determined after the diagnostic work-up of patients, including a US carotid examination, echocardiogram, or a 24-h electrocardiogram.

### Carotid MR imaging protocol

To define the symptomatic carotid artery, all patients underwent a routine brain MRI scan and carotid ultrasonographic screen. Then, they were performed on a 3 T MR scanner with 8-channel phase array carotid coils [12]. A standardized multisequence protocol was performed for the carotid artery bifurcation on the symptomatic side by acquiring three-dimensional time of flight (3D-TOF), T1-weighted (T1W), T2-weighted (T2W), and magnetization-prepared rapid acquisition gradient echo (MP-RAGE) sequences [12]. The contrast material was gadolinium diethylenetriamine pentametric acid (GD-DTPA) at an injection dose of 0.1 mmol/Kg. In addition, delayed T1WI scanning was performed for about 5 minutes after intravenous injection. Finally, spectral preservation attenuated inversion recovery (SPAIR) was used in the black blood technique to enhance the contrast between the vessel wall and the surrounding tissue. Therefore, the total scanning time was about 35 minutes. The imaging parameters are as follows [12], (1) 3D-TOF MRA: repetition Time (TR)/echo Time (TE) = 20/4.9 ms, Flip Angle (FA) = 20°; Field of view (FOV) = 140 × 140 mm, matrix size = 256 × 256, slice thickness = 1.0 mm; (2) T1W: TR/TE = 800/10 ms, FA = 90°, FOV = 140 mm × 140 mm, Matrix size = 256 × 256, slice thickness = 2.0 mm; (3) T2W: TR/TE = 4800/50 ms, FA = 90°; FOV = 140 mm × 140 mm, matrix size = 256 × 256, slice thickness = 2.0 mm; (4)MP-RAGE: TR/TE = 8.8/5.3 ms, FA = 15°, FOV = 140 mm × 140 mm, Matrix size = 256 × 256, slice thickness = 1.0 mm.

### MRI image analysis

The custom-designed software CASCADE (Vascular Imaging Lab, University of Washington) [20] was used to review the MR vessel wall images of carotid arteries [12]. The lumen and outer vessel boundaries were outlined manually on T2 -weighted images. Then, the lumen area, wall area, total vessel area, and maximum wall thickness were automatically calculated. The reference values were defined as the normal vessels located in the contralateral portion or proximal to the stenotic position. The parameters of plaque were defined as follows: 1) wall area (WA) = vessel area (VA) − lumen area (LA); 2) remodeling index (RI) = vessel area at stenotic lesion/ vessel area at reference vessel. The presence of calcification, lipid-rich necrotic core (LRNC), and IPH were detected and measured quantitatively using the published criteria [21, 22]. In particular, IPH was demonstrated by a hyperintense region in the plaque on TOF, T1W, and MP-RAGE sequence images. Meanwhile, the volumes, percent of wall volume, and component volumes were calculated from the measured areas according to the published criteria [12]. If there were multiple plaques in the symptomatic artery, the plaque leading to the highest degree of stenosis was selected for analysis [23]. The luminal stenosis of carotid arteries was measured using the algorithm of North American Symptomatic Carotid Endarterectomy Trial Collaborators (NASCET) [4] and was divided into 0, 1 to 29%, 30 to 49%, and ≥ 50% categories [12]. The image quality was divided into four grades [24]: grade 1 = the image of the carotid lumen and the outside of the blood vessel was not clear, or the signal-noise ratio (SNR) was low, and there were noticeable motion artifacts; Grade 2 = the carotid artery wall was visible in the image, but the wall structure and the external contour of the vessel could not be judged, and SNR was low, or there were motion artifacts; Grade 3 = the carotid artery wall structure, lumen, and outside vascular contour were clear, the blood flow signal in the lumen was not wholly suppressed, and SNR was high, accompanied by a small amount of movement or swallowing artifacts; Grade 4 = the carotid artery wall structure, lumen, and outside vascular contour were clear, the blood flow signal in the lumen was wholly suppressed, and SNR was high without apparent artifacts. Therefore, the image quality of≤grade 2 was excluded from the statistical analysis. Two experienced senior neuroimaging physicians reviewed all slices of the sequences of carotid plaques on HR VWMRI, blinded to the clinical information and the diffusion-weighted images (DWI). Consensus interpretation was accepted for the final analysis if the two readers’ interpretations were different. According to the reproducibility test, twenty patients were randomly selected from the study population every 2 months to test the consistency of inter-reader and intrareader in measuring carotid plaque morphology and carotid plaque compositions, and the final database was accepted if intraclass correlation coefficients (ICC) > 0.80 [23].

### Outcome and follow-up

Patients or their authorized proxies were visited by telephone interview at 3, 6, 9, and 12 months after discharge. The primary outcome was the recurrence of ipsilateral ischemic stroke, defined as a sudden onset of focal neurological deficit lasting > 24 hours in the ipsilateral carotid territory [25]. The terminating points were TIA, carotid endarterectomy (CEA)/carotid artery stenting (CAS), or death [26]. All recurrent ischemic events were confirmed according to the clinical medical records, which were defined as ischemic stroke, TIA, acute coronary syndrome, and peripheral artery disease [26]. Two experienced stroke neurologists reviewed patients’ medical documents to ensure a reliable diagnosis of recurrent ischemic stroke. If the patient’s information was missing or the patient died without hospitalization, two neurologists made the decision based on the medical documents. Any death of patients was confirmed by checking the hospital medical records or local citizen registration. In addition, the following events were also recorded over the entire follow-up period: 1) new atrial fibrillation; 2) other stroke (contralateral stroke/posterior circulation stroke); 3) other vascular events (cardiovascular events/peripheral vascular events); 4) death from non-vascular events; 5) hemorrhage. Two trained stroke neurologists in Beijing Tian Tan hospital completed all the telephone follow-ups of patients.

### Statistical analysis

Continuous variables were described by means (standard deviations [SDs]) or medians (interquartile ranges [IQRs]), and categorical data were expressed as frequency and percentage. Cohen’s kappa coefficient and ICC were assessed for inter-reader and intrareader reproducibility. Differences between the IPH and non-IPH groups were analyzed using Student’s t-test or Mann–Whitney U- test for continuous variables according to a normal distribution and chi-squared test or Fisher exact test for categorical variables. By calculating hazard ratio (HR) and 95% confidence interval (CI), we used multivariable Cox regression models to study predictors of stroke recurrence with a *P* < 0.05 and parameters with clinical significance in univariate analysis. Kaplan-Meier survival analysis and a log-rank test were used to compare recurrence-free survival time with the IPH and non-IPH groups. Two-sided tests were used for all statistical data, with a *P* < 0.05 considered statistically significant. All statistical analysis was conducted by the SPSS version 22.0 (IBM, NY, USA).

## Results

**Fig. 1:**
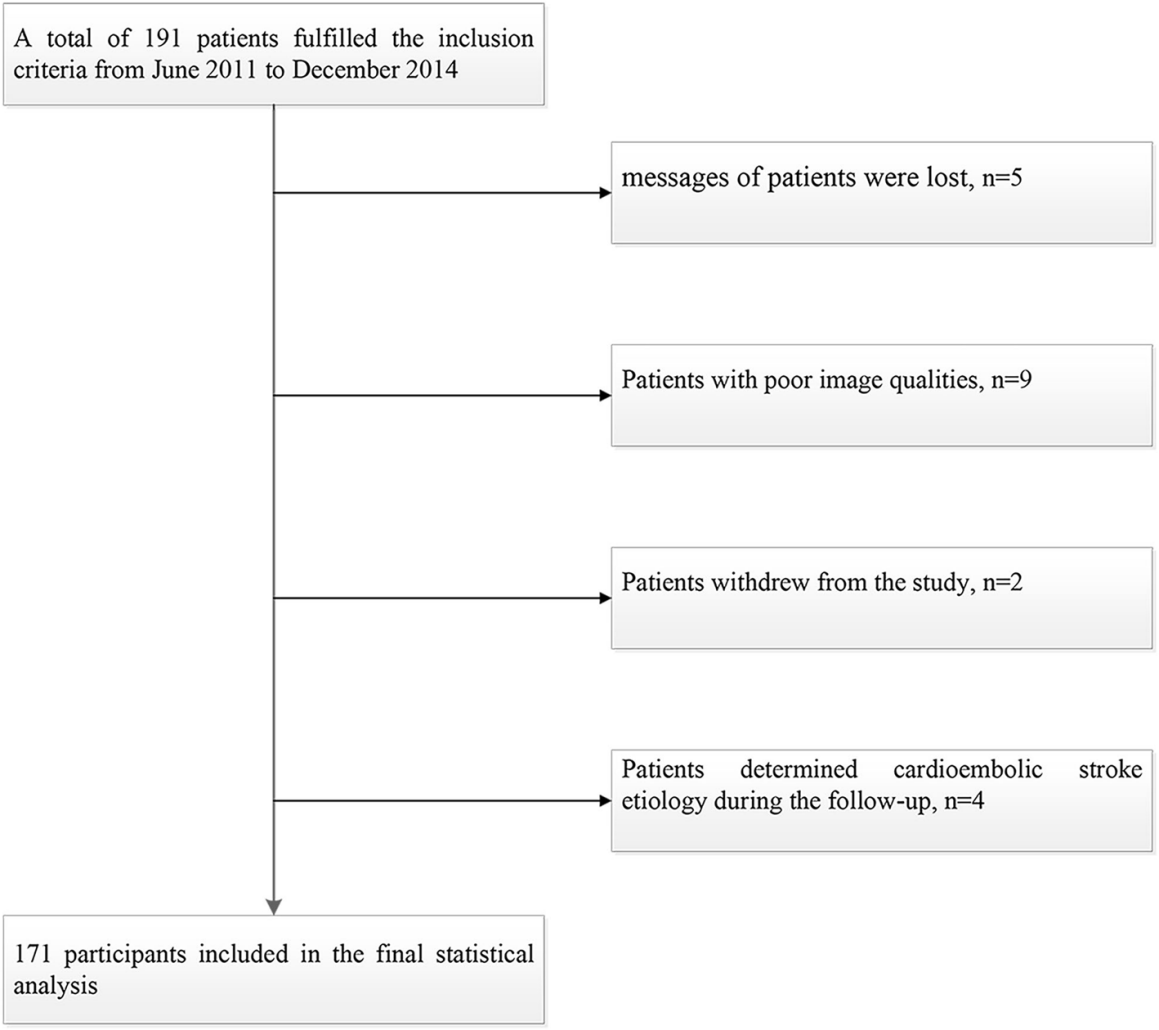
Flow chart of study patients

**Fig. 2:**
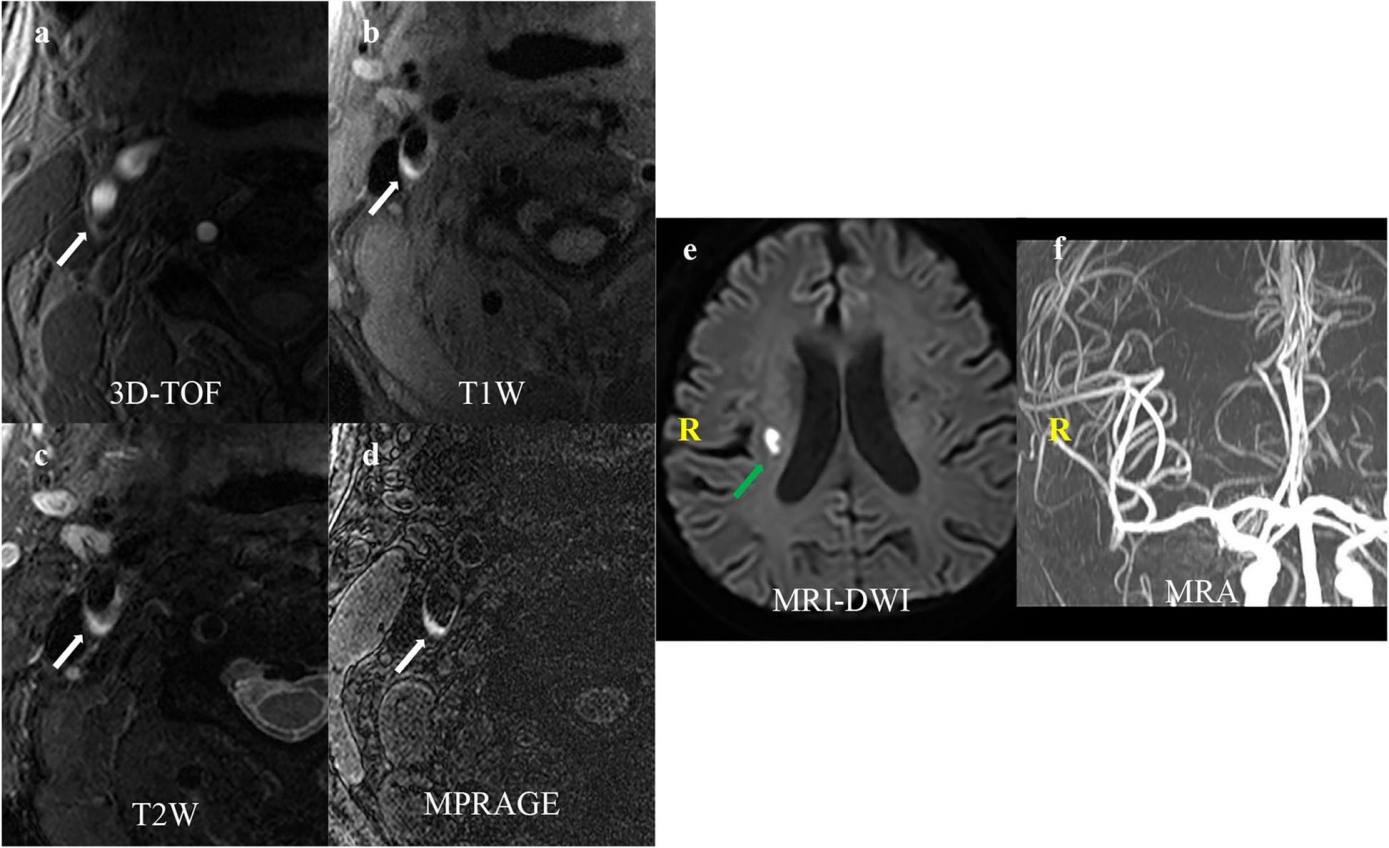
A 64-year-old male patient presented with a right ischemic stroke. IPH is demonstrated by the hyperintensity on (**a**) 3D-TOF, (**b**) T1W, (**d**) MPRAGE, and hyperintensity on (**c**) T2W (white arrow). There is a hyperintensity lesion in the blood-supply area of the right carotid artery on (**d**) DWI (green arrow) and no evident ipsilateral intracranial vascular stenosis or occlusion on (**e**) MRA. DWI, diffusion-weighted images; MRA, magnetic resonance artery; 3D-TOF, three-dimensional time of flight; T1W, T1-weighted; T2W, T2-weighted; MPRAGE, magnetization-prepared rapid acquisition gradient-echo

A total of 191 patients were enrolled from June 2011 to December 2014, and 20 patients were excluded due to the following reasons: 1) the clinical messages of 5 patients were lost; 2) 9 patients with poor image qualities; 3) 2 patients withdrew from the study; 4) 4 patients determined cardioembolic stroke etiology during the follow-up. Thus, a total of 171 patients (mean age, 59.66 ± 10.06 years) were included in the final statistical analysis, including 118 males (69.0%) (Fig. 1). The mean follow-up time was (10.39 ± 2.66) months. During the follow-up, 17 patients underwent CEA/CAS, and 11 patients suffered recurrent neurological events before undergoing CAS. Meanwhile, twenty-eight patients (16.4%) occurred ipsilateral cerebral ischemic events, including twenty-three patients (13.5%) with ipsilateral cerebral stroke and five patients (2.9%) with TIA. Intraplaque hemorrhage detected by HR VWMRI was found in 32 of 171 patients (Fig. 2).

There were no significant differences in gender, hypertension, hyperlipidemia, coronary heart disease, prior stroke/TIA, family history of stroke, alcohol consuming, serum cholesterol (TC), triglycerides (TG), low-density lipoprotein cholesterol (LDL-C), high-density lipoprotein cholesterol (HDL-C) between the IPH group and the non-IPH group. However, there were significant differences in age (mean, 64.50 ± 9.60 vs 59.13 ± 9.90, *P* = 0.006), diabetes mellitus (43.8% vs 25.9%, *P* = 0.045), smoking history (68.8% vs 46.8%, *P* = 0.025), NIHSS on admission (3 [0–8] vs 5 [0–18], *P* = 0.004), pre-admission mRS (1 [0–3] vs 1 [0–5], *P* = 0.030), hs-CRP (7.71 [0.10–17.90] mg/L vs 3.72 (0.00–31.90) mg/L, *P* = 0.002), and hcy (18.99 [9.20–75.00] umol/L vs 14.10 [1.11–41.20] umol/L, *P* = 0.028) between the two groups (Table 1). And Multivariate Logistic regression analysis showed that the factors associated with IPH were age (OR,1.06, 95% CI, 1.01–1.11; *P* = 0.11), Hcy (OR,1.06, 95% CI, 1.01–1.11; *P* = 0.012), hs-CRP (OR,1.09, 95% CI, 1.01–1.12; *P* = 0.022) (Table 1).

**Table 1:** Demographic and clinical characteristics in patients

Univariate analysis				
Variables	Total (***n*** = 171)	IPH group (***n*** = 32)	Non-IPH group (***n*** = 139)	***P***-Value
Gender, male, n (%)	118 (69.0)	25 (78.1)	93 (66.9)	0.216
Age, years	60.13 ± 10.04	64.50 ± 9.60	59.13 ± 9.90	0.006
Hypertension, n (%)	116 (67.8)	22 (68.8)	94 (67.6)	0.902
Diabetes mellitus, n (%)	50 (29.2)	14 (43.8)	36 (25.9)	0.045
Coronary heart disease, n (%)	20 (11.7)	4 (12.5)	16 (11.5)	0.876
Hyperlipidemia, n (%)	56 (32.7)	6 (18.8)	50 (36.0)	0.061
Prior stroke/TIA, n (%)	27 (15.8)	5 (15.6)	22 (15.8)	0.977
Family history of stroke, n (%)	24 (14.0)	7 (21.9)	17 (12.2)	0.165
Smoking history, n (%)	87 (50.9)	22 (68.8)	65 (46.8)	0.025
Alcohol consuming, n (%)	56 (32.7)	10 (31.3)	46 (33.1)	0.841
NIHSS on admission, IQR	4 (0–18)	3 (0–8)	5 (0–18)	0.004
Pre-admission mRS, IQR	1 (0–5)	1 (0–3)	1 (0–5)	0.030
hs-CRP, IQR, mg/L	4.28 (0.00–31.90)	7.71 (0.10–17.90)	3.72 (0.00–31.90)	0.002
TC, mmol/L	4.35 ± 1.08	4.24 ± 1.02	4.38 ± 1.10	0.535
TG, IQR, mmol/L	1.32 (0.49–8.44)	1.14 (0.67–2.29)	1.31 (0.49–4.70)	0.190
LDL-C, mmol/L	2.64 ± 0.97	2.54 ± 0.99	2.66 ± 0.97	0.545
HDL-C, IQR, mmol/L	1.01 (0.48–1.71)	0.99 (0.64–1.39)	1.01 (0.48–1.71)	0.605
Hcy, IQR, umol/L	14.90 (1.11–75.00)	18.99 (9.20–75.00)	14.10 (1.11–41.20)	0.028
Median enrolled-to-HRVMRI scan time, IQR, d	4 (2–6)	4 (2–6)	5 (2–6)	0.182
Previous use of aspirin, n (%)	33 (19.3)	6 (18.8)	27 (19.4)	0.931
Previous use of Statins, n (%)	26 (15.2)	4 (12.5)	22 (15.8)	0.788
Taking aspirin until primary outcome/Terminating points, n (%)	95 (55.6)	19 (59.4)	76 (54.7)	0.630
Taking statins until primary outcome/Terminating points, n (%)	71 (41.5)	10 (31.3)	61 (43.9)	0.191
Percentage of stenosis degree, IQR, %	35 (4–58)	31.10 (4–43)	36.35 (6–58)	< 0.001
Categories of stenotic degree				0.031
< 30%, n (%)	50 (29.2)	15 (46.9)	35 (25.2)	
30–49%, n (%)	112 (65.5)	17 (53.1)	95 (68.3)	
> 50%, n (%)	9 (5.3)	0 (0)	9 (6.5)	
Plaque burden				
LA, IQR, mm^2^	16.39 (2.06–42.38)	14.30 (2.64–31.96)	16.67 (2.06–42.38)	0.040
WA, IQR, mm^2^	54.81 (28.03–160.57)	73.58 (33.10–160.57)	53.22 (28.03–116.38)	< 0.001
VA, IQR, mm^2^	74.84 (43.00–162.11)	73.98 (43.00–162.11)	74.84 (45.01–140.50)	0.244
NWI, IQR	0.56 (0.40–0.97)	0.69 (0.53–0.97)	0.53 (0.40–0.94)	< 0.001
WT, IQR, mm	2.88 (1.00–11.35)	4.40 (3.07–11.35)	2.65 (1.00–7.56)	< 0.001
PA, IQR, mm^2^	24.51 (5.61–74.29)	29.90 (14.24–71.35)	22.93 (5.61–74.29)	0.001
RI	0.75 ± 0.08	0.72 ± 0.08	0.75 ± 0.08	0.012
Plaque component				
LRNC				
Presence, n (%)	139 (81.3)	32 (100)	107 (77.0)	0.003
Volume, IQR, mm^3^	35.70 (0–660.22)	176.17 (2.86–660.22)	26.30 (0–352.34)	< 0.001
Calcification				
Presence, n (%)	94 (55.5)	26 (81.3)	68 (48.9)	0.001
Volume, IQR, mm^3^	2.12 (0–290.56)	19.04 (0–290.56)	0.00 (0–253.90)	< 0.001
Loose matrix				
Presence, n (%)	79 (46.2)	21 (65.6)	58 (41.7)	0.014
Volume, IQR, mm^3^	0 (0–95.46)	12.05 (0–86.54)	0.00 (0.00–95.46)	< 0.001
FCR, n (%)	6 (3.5)	0 (0)	6 (0.7)	0.596
Primary outcome				
ipsilateral ischemic stroke	23 (13.5)	14 (43.8)	9 (6.5)	–
Terminating points				
TIA	5 (2.9)	3 (9.4)	2 (1.4)	–
CEA/CAS	17 (9.9)	3 (9.4)	14 (10.1)	–

Abbreviation: *IPH* intraplaque hemorrhage, *TIA* transient ischemic attack, *NIHSS* national institutes of health stroke scale, *mRS* modified Rankin scale, *IQR* interquartile range, *SBP* systolic blood pressure, *DBP* diastolic blood pressure, *hs-CRP* hypersensitive c-reactive protein, *TC* cholesterol, *TG* triglycerides, *LDL-C* low-density lipoprotein cholesterol, *HDL-C* high-density lipoprotein cholesterol, *Hcy* homocysteine, *CA* carotid artery, *LA* lumen area, *WA* wall area, *VA* vessel area, *NWI* normalized wall index, *WT* wall thickness, *PA* plaque area, *RI* remodeling index, *LRNC* lipidlipo-rich necrotic core, *FCR* fibrous cap rupture, *CEA* carotid endarterectomy, *CAS* carotid artery stenting

Between the IPH group and the non-IPH group, there was a significant difference in the percentage of stenosis degree (31.10 [4–43]% vs 36.35 [6–58]%，*P* < 0.001). There was no significant difference in vessel area (VA) between the two groups (*P* = 0.244 > 0.05). However, there were significant differences in the lumen area (LA) (14.30 [2.64–31.96] mm^2^ vs 16.67 [2.06–42.38] mm^2^, *P* = 0.040), wall area (WA) (73.58 [33.10–160.57] mm^2^ vs 53.22 [28.03–116.38] mm^2^, *P* < 0.001), normalized wall index (NMI) (0.69 [0.53–0.97] vs 0.53(0.40–0.94), *P* < 0.001), wall thickness (WI) (4.40 [3.07–11.35] mm vs 2.65 (1.00–7.56) mm, *P* < 0.001), plaque area (PA) (29.90 [14.24–71.35] mm^2^ vs 22.93 [5.61–74.29] mm^2^, *P* = 0.001), and remodeling index (RI) (0.72 ± 0.08 vs 0.75 ± 0.08, *P* = 0.012). At the same time, there were significant differences in the presence of plaque components between the two groups, including calcification (81.3% vs 48.9%, *P* = 0.001), loose matrix (65.6% vs 58.0%, *P* = 0.014), and LRNC (100% vs 77.0%, *P* = 0.003) (Table 1).

Univariate Cox regression analysis showed that several factors were associated with recurrent stroke, including age (HR, 1.05, 95% CI, 1.01–2.00; *P* = 0.029), taking aspirin until primary outcome (HR, 0.38, 95% CI, 0.16–0.89; *P* = 0.027), taking statins until primary outcome (HR, 0.27, 95% CI, 0.09–0.78; *P* = 0.016), and the plaque characteristics such as loose matrix (HR, 4.50, 95% CI, 1.60–12.11; *P* = 0.003), LRNC (HR, 2.73, 95% CI, 1.21–6.19; *P* = 0.016)，and IPH (HR, 6.40, 95% CI, 2.77–14.82; *P* < 0.001) (Table 2).

**Table 2:** Univariate and Multivariate Cox regression analysis for predictors of ipsilateral stroke recurrence at 12 months

Variables	Univariate Cox regression			Multivariate Cox regression		
	HR	95%CI	***P*** value	HR	95%CI	***P*** value
Age, years	1.05	1.01–2.00	0.029	–	–	0.957
Taking aspirin until primary outcome	0.38	0.16–0.89	0.027	0.24	0.10–0.60	0.002
Taking statins until primary outcome	0.27	0.09–0.78	0.016	–	–	0.275
Loose matrix	4.50	1.6–12.11	0.003	4.08	1.48–11.24	0.007
LRNC	2.73	1.21–6.19	0.016	–	–	0.456
IPH	6.40	2.77–14.82	< 0.001	8.68	3.62–20.85	< 0.001

Abbreviation: *HR* hazard ratio, *CI* confidence interval, *LRNC* lipidlipo-rich necrotic core, *IPH* intraplaque hemorrhage

And Multivariate Cox regression analysis showed that the factors associated with recurrent stroke were loose matrix (HR, 4.08, 95% CI, 1.48–11.24; *P* = 0.007) and IPH (HR, 8.68, 95% CI, 3.62–20.85; *P* < 0.001). However, the medication of aspirin used until the primary outcome was the protective factor of recurrent stroke (HR, 0.24, 95% CI, 0.10–0.60; *P* = 0.002) (Table 2).

After adjusting for confounding factors (age, gender, NIHSS on admission, pre-admission mRS, hypertension, diabetes mellitus, coronary heart disease, hyperlipidemia, previous history of stroke, smoking history, and family history of stroke, stenotic degree, aspirin used for 1 year, statins used for 1 year, plaque calcification, loose matrix, lipid-rich necrotic core, and plaque fibrous cap rupture), the prevalence of IPH still could predict recurrent ischemic events (adjusted HR, 6.64, 95% CI, 2.84–15.54; *P* < 0.001) and ipsilateral recurrent ischemic stroke (adjusted HR,8.08, 95% CI, 3.65–17.91, *P* < 0.001) (Table 3).

**Table 3:** Analysis of cerebral ischemic events in patients with IPH

	HR	95%CI	***P*** value
**Ipsilateral recurrent stroke**			
Model 1^a^	8.10	3.50–18.74	< 0.001
Model 2^b^	6.88	2.94–16.09	< 0.001
Model 3^c^	6.64	2.84–15.54	< 0.001
**Ipsilateral recurrent stroke, TIA**			
^+^Model 1^a^	8.36	3.91–17.87	< 0.001
^+^Model 2^b^	7.35	3.40–15.91	< 0.001
^+^Model 3^c^	8.08	3.65–17.91	< 0.001

^a^ Model 1 adjusted for age, gender, NIHSS on admission, pre-admission mRS.

^b^ Model 2 further adjusted for hypertension, diabetes mellitus, coronary heart disease, hyperlipidemia, previous history of stroke, smoking history, and family history of stroke

^c^ Model 3 further adjusted for stenotic degree, taking aspirin until primary outcome, taking statins until primary outcome, plaque calcification, loose matrix, lipo-rich necrotic core, and plaque fibrous cap rupture

^+^ Cox regression analysis on the association IPH with ipsilateral recurrent stroke, TIA

**Fig. 3:**
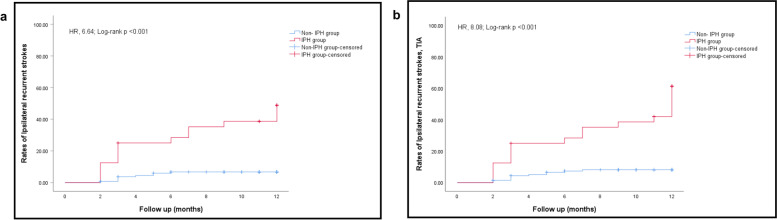
Survival analysis (Kaplan–Meier plot) figures confirm the predictive value of IPH for (**a**) stroke and (**b**) all cerebral ischemic events. The x-axis represents the time of follow-up in months. The y-axis represents the proportion of patients who had ipsilateral stroke recurrence. IPH, intraplaque hemorrhage; HR, hazard ratio

In the IPH group, 14 patients suffered recurrent ipsilateral strokes, and three patients suffered TIA (Table 1). Kaplan–Meier analysis showed that IPH was associated with ipsilateral recurrent stroke (*P* < 0.001; Fig. 3a) and recurrent cerebral ischemic events at 12 months compared to the non-IPH group (*P* < 0.001; Fig. 3b).

## Discussion

Our study investigated the association between IPH and ipsilateral recurrent ischemic stroke. Schindler et al. [13] found that carotid IPH could be more than 50% in patients with symptomatic carotid stenosis. However, the incidence of carotid IPH in our study was 18.71% (32 of 171). We considered that the proportion of patients with mild stenosis in our study was much higher than in other studies, and only nine patients had a degree of stenosis > 50% in our study. Saam et al. [27] found that most atherosclerotic carotid lesions defined by American Heart Association type VI (AHA-LT6) were 37.0%, while the incidence of IPH was 84.4%. Subsequently, Saam et al. [28] found that the incidence of IPH was as high as 49.0% in symptomatic carotid atherosclerosis patients in a meta-analysis.

Some studies have confirmed the strong correlation between IPH and acute stroke [26, 29]. However, there were seldom studies on the relationship between IPH and neurological deficit. Our study found a significant difference in NIHSS on admission (*P* = 0.004) between the IPH and non-IPH groups. Still, patients with IPH might have less neurological impairment than patients without IPH. We don’t know the reason for the result, and it would be a meaningful research focus.

Concerning the risk factors for IPH, some studies have found that the prevalence of carotid IPH would increase with age increasing and was associated with multiple cardiovascular risk factors (e.g., advanced age, male sex, presence of carotid stenosis, HDL) [30, 31]. Nevertheless, Catalano et al. [32] analyzed atherosclerotic burden and plaque composition quantitatively and found that a history of hypercholesterolemia, diabetes, hypertension or smoking did not correlate with IPH.

Our study found that the factors such as age, Hcy, and hs-CRP were associated with IPH, and the combination of such risk factors might predict the existence of IPH. Due to the limitations of our study, we did not conduct further research. We hope that future extensive sample studies can explore the predictors of IPH to implement intervention measures as soon as possible.

Some studies have confirmed that the prevalence of IPH is associated with plaque morphology and degree of stenosis [33–35], which is also found in our research. We found that the patients with IPH might have more severe stenosis of the carotid artery, larger plaque area, more oversized WI, and less RI. At the same time, IPH might be related to the plaque components such as calcification, loose matrix, and LRNC. Because of the limited research ability, we had no further study on this result, and it would be our research direction in the future.

Although some studies have confirmed that the degree of carotid stenosis is associated with stroke recurrence [4], it is not sufficient to accurately predict the risk of stroke recurrence [36]. Our study did not find that carotid stenosis was associated with stroke recurrence owing to the lower percentage of patients with stenosis (> 50%), which was only 5.3% (9 of 171).

About 20% of patients with first ischemic stroke would undergo recurrent ischemic vascular events within 1 year [37]. Studies have confirmed that IPH was significantly associated with stroke recurrence [38, 39]. Deng, F et al. [40] also confirmed that the presence of IPH in carotid plaque, LRNC, and FCR shown on MRI were strong predictors of recurrent stroke events in a recent meta-analysis; the hazard ratios were 7.14 (95%CI, 4.32–11.82), 5.68 (95%CI, 2.40–13.47), and 2.73 (95%CI, 1.04–7.16), respectively. In our study, 13.5% of patients with carotid atherosclerosis occurred ipsilateral cerebral stroke, and 16.4% occurred ischaemic cerebrovascular events (stroke and TIA). The recurrence rate of an ipsilateral stroke in patients with IPH could be 6.30 times higher than in patients without IPH even after adjusting for multiple cardiovascular risk factors, medication (taking aspirin or statins), and other vulnerable plaque features (such as calcification, loose matrix, LRNC, and FCR).

By Kaplan–Meier survival analysis, we found the estimated probability of recurrent cerebrovascular stroke in patients with IPH was 25.0% at 3 months, 28.4% at 6 months, 38.6% at 9 months, and 48.9% at 12 months. However, the risk of stroke in patients without IPH was only 3.66% at 3 months and 6.68% at 12 months. Some previous studies have confirmed that recurrent cerebrovascular events often occur within 14–90 days after the index stroke [41, 42]. Such results give us a hint that we might take more active and effective interventions on patients with acute ischemic stroke as soon as possible.

Our study found that taking aspirin or statins was the protective factor for stroke recurrence. We also tried to determine whether it was also a protective factor for IPH, which has been confirmed in some studies [43, 44]. Unfortunately, our results were negative. We thought it might be due to the low proportion of taking aspirin or statins in patients with vulnerable carotid plaque. We believe it would be our future research direction.

There are some shortcomings in this study. Firstly, it is well known that atherosclerotic disease is a persistent chronic lesion and will accelerate deterioration with the increasing of time and age. However, we did not detect the change of carotid atherosclerotic plaques in patients accepting standardized secondary prevention strategies for stroke. Secondly, due to the limitations of the funds, we only studied plaques in the extracranial carotid artery carotid bifurcation. We did not exclude the interfering factors of plaques in other parts of extracranial carotid arteries and intracranial arteries, which may lead to errors in the results. Thirdly, we included a sufficient number of variables in the multivariate analysis, and the statistical results might be inevitably biased due to the small sample size.

Nevertheless, our study still put some insight into the future. Firstly, we may need to implement more active and effective intervention strategies for patients with IPH. Secondly, the future research hot point may explore the pathophysiological mechanism of plaque vulnerability and the molecular biomarkers combined with neuroimaging markers for risk stratification of recurrent ischemic events in symptomatic patients.

## Conclusions

Compared with non-IPH, the patients with IPH have a higher risk of 1-year ischemic stroke recurrence. The potential risk of IPH should not be overlooked. It will be the future research hot point to understand the underlying pathophysiological mechanism of plaque vulnerability and the molecular biomarkers combined with neuroimaging markers.

## Supplementary Information

**Additional file 1.**

## Data Availability

The datasets generated and/or analysed during the current study are not publicly available due to the reason that there is a potential for disclosure of patient information but are available from the corresponding author on reasonable request.

## Abbreviations

IPH: Intraplaque hemorrhage
TIA: Transient ischemic attack
NIHSS: National institutes of health stroke Scale
mRS: Modified rankin scale
IQR: Interquartile range
SBP: Systolic blood pressure
DBP: Diastolic blood pressure
hs-CRP: Hypersensitive c-reactive protein
TC: Cholesterol
TG: Triglycerides
LDL-C: Low-density lipoprotein cholesterol
HDL-C: High-density lipoprotein cholesterol
Hcy: Homocysteine
CA: Carotid artery
LA: Lumen area
WA: Wall area
VA: Vessel area
NWI: Normalized wall index
WT: Wall thickness
PA: Plaque area
RI: Remodeling index
LRNC: Lipid-rich necrotic core
FCR: Fibrous cap rupture
CEA: Carotid endarterectomy
CAS: Carotid artery stenting
OR: Odds ratio
CI: Confidence interval
HR: Hazard ratio
CI: Confidence interval
LRNC: Lipid-rich necrotic core
IPH: Intraplaque hemorrhage
