# Supplementary material for: Extracranial carotid plaque hemorrhage predicts ipsilateral stroke recurrence in patients with carotid atherosclerosis – a study based on high-resolution vessel wall imaging MRI
Source: BMC Neurol. 2022 Jun 28;22:237. doi: 10.1186/s12883-022-02758-3 (PMC9238155; doi:10.1186/s12883-022-02758-3)
Supplement: Supplementary file 1 — Additional file 1. [file 12883_2022_2758_MOESM1_ESM.zip › Supplementary/C2-revised Supplementary 2- result of cox regression.doc.docx]

GET

FILE='D: \ Che Fengli is important!!!\ CCA raw data \ AA-CCA-IPH related factors \ Aa Che-survival status.sav-2022-2-25.sav'.

DATASET NAME dataset 1 WINDOW=FRONT.

COXREG survevalmonth

/STATUS=outcome2('1') /CONTRAST(gender)=Indicator

/METHOD=ENTER age_01 gender preaddmisionmRS_01 NIHSS_A IPH1

/PRINT=CI(95)

/CRITERIA=PIN(.05) POUT(.10) ITERATE(20).

Cox regression

| **remarks** | | |
| --- | --- | --- |
| The output has been created | | 22-MAY-2022 11:14:12 |
| explanatory note | |  |
| import | data | D: \ Che Fengli is important!!!\ CCA raw data \ AA-CCA-IPH related factors \ Aa Che-survival status.sav-2022-2-25.sav |
|  | Activity dataset | data set 1 |
|  | filter | <not have> |
|  | weight | <not have> |
|  | Split files | <not have> |
|  | Number of rows in the working data file | 171 |
| Missing value processing | Definition of deletions | Treat the user-defined missing values as missing. |
| grammar | | COXREG survevalmonth  /STATUS=outcome2('1') /CONTRAST(gender)=Indicator  /METHOD=ENTER age_01 gender preaddmisionmRS_01 NIHSS_A IPH1  /PRINT=CI(95)  /CRITERIA=PIN(.05) POUT(.10) ITERATE(20). |
| resource | Handler time | 00:00:00.02 |
|  | It takes time | 00:00:00.01 |

[Data set 1] D: \ Che Fengli is important!!!\ CCA raw data \ AA-CCA-IPH related factors \ Aa Che-survival status.sav-2022-2-25.sav

| **Case handling summary** | | | |
| --- | --- | --- | --- |
|  | | The number of cases | percentage |
| Cases that can be used in the analysis | event^a^ | 28 | 16.4% |
|  | After checking out | 141 | 82.5% |
|  | amount to | 169 | 98.8% |
| Deleted cases | Cases with missing values | 2 | 1.2% |
|  | Cases with a negative time period | 0 | 0.0% |
|  | The first incident in the layer | 0 | 0.0% |
|  | amount to | 2 | 1.2% |
| amount to | | 171 | 100.0% |

| a.Dependent variable: survevalmonth |
| --- |

| **Classification variable coding^a^** | | | |
| --- | --- | --- | --- |
|  | | frequency | (1) |
| A Gender: 0= female; 1= male^b^ | 0=female | 52 | 1 |
|  | 1=male | 117 | 0 |

| a.Category variable: ^ 1 (gender) |
| --- |
| b.Indicator parameter encoding |

Block 0: Start block

| **The Omnibus's test of the model coefficients** |
| --- |
| -2 log-likelihood |
| 274.973 |

Block 1: Method = Input

| **The Omnibus's test of the model coefficients^a^** | | | | | | | | | |
| --- | --- | --- | --- | --- | --- | --- | --- | --- | --- |
| -2 log-likelihood | Overall (score) | | | Make the changes from before | | | Change it from the previous block |  |  |
|  | chi-square | free degree | conspicuousness | chi-square | free degree | conspicuousness | chi-square |  |  |
| 243.096 | 45.024 | 5 | .000 | 31.876 | 5 | .000 | 31.876 |  |  |

| **Variables in Eq** | | | | | | | | |
| --- | --- | --- | --- | --- | --- | --- | --- | --- |
|  | B | SE | Wald | free degree | conspicuousness | Exp(B) |  |  |
|  |  |  |  |  |  |  |  |  |
| A age | .012 | .020 | .385 | 1 | .535 | 1.012 |  |  |
| A Gender: 0= female; 1= male | -.315 | .467 | .456 | 1 | .500 | .729 |  |  |
| Pre-addmision mRS | -.273 | .264 | 1.072 | 1 | .300 | .761 |  |  |
| A7. Baseline NIHSS | .013 | .091 | .020 | 1 | .886 | 1.013 |  |  |
| A IPH | 1.936 | .421 | 21.113 | 1 | .000 | 6.932 |  |  |

| **Mean values of covariates** | |
| --- | --- |
|  | average value |
| A age | 60.189 |
| A Gender: 0= female; 1= male | .308 |
| Pre-addmision mRS | 1.408 |
| A7. Baseline NIHSS | 4.793 |
| A IPH | .178 |

COXREG survevalmonth

/STATUS=outcome2('1') /CONTRAST(gender)=Indicator

/METHOD=FSTEP(LR) age_01 gender preaddmisionmRS_01 NIHSS_A IPH1

/PRINT=CI(95)

/CRITERIA=PIN(.05) POUT(.10) ITERATE(20).

Cox regression

| **remarks** | | |
| --- | --- | --- |
| The output has been created | | 22-MAY-2022 11:15:10 |
| explanatory note | |  |
| import | data | D: \ Che Fengli is important!!!\ CCA raw data \ AA-CCA-IPH related factors \ Aa Che-survival status.sav-2022-2-25.sav |
|  | Activity dataset | data set 1 |
|  | filter | <not have> |
|  | weight | <not have> |
|  | Split files | <not have> |
|  | Number of rows in the working data file | 171 |
| Missing value processing | Definition of deletions | Treat the user-defined missing values as missing. |
| grammar | | COXREG survevalmonth  /STATUS=outcome2('1') /CONTRAST(gender)=Indicator  /METHOD=FSTEP(LR) age_01 gender preaddmisionmRS_01 NIHSS_A IPH1  /PRINT=CI(95)  /CRITERIA=PIN(.05) POUT(.10) ITERATE(20). |
| resource | Handler time | 00:00:00.02 |
|  | It takes time | 00:00:00.01 |

| **Case handling summary** | | | |
| --- | --- | --- | --- |
|  | | The number of cases | percentage |
| Cases that can be used in the analysis | event^a^ | 28 | 16.4% |
|  | After checking out | 141 | 82.5% |
|  | amount to | 169 | 98.8% |
| Deleted cases | Cases with missing values | 2 | 1.2% |
|  | Cases with a negative time period | 0 | 0.0% |
|  | The first incident in the layer | 0 | 0.0% |
|  | amount to | 2 | 1.2% |
| amount to | | 171 | 100.0% |

| a.Dependent variable: survevalmonth |
| --- |

| **Classification variable coding^a^** | | | |
| --- | --- | --- | --- |
|  | | frequency | (1) |
| A Gender: 0= female; 1= male^b^ | 0=female | 52 | 1 |
|  | 1=male | 117 | 0 |

| a.Category variable: ^ 1 (gender) |
| --- |
| b.Indicator parameter encoding |

Block 0: Start block

| **Variables not included in Eq^a^** | | | |
| --- | --- | --- | --- |
|  | score | free degree | conspicuousness |
| A age | 2.045 | 1 | .153 |
| A Gender: 0= female; 1= male | 1.316 | 1 | .251 |
| Pre-addmision mRS | 4.726 | 1 | .030 |
| A7. Baseline NIHSS | 4.188 | 1 | .041 |
| A IPH | 42.959 | 1 | .000 |

| a.Residual chi-square = 45.024, degree of freedom of 5, significance =.000 |
| --- |

Block 1: Method = Step forward (likelihood ratio)

| **The Omnibus's test of the model coefficients^b^** | | | | | | | | | | |
| --- | --- | --- | --- | --- | --- | --- | --- | --- | --- | --- |
| step | -2 log-likelihood | Overall (score) | | | Make the changes from before | | |  |  |  |
|  |  | chi-square | free degree | conspicuousness | chi-square | free degree | conspicuousness |  |  |  |
| 1^a^ | 245.693 | 42.959 | 1 | .000 | 29.280 | 1 | .000 |  |  |  |

| **Variables in Eq** | | | | | | | | | |
| --- | --- | --- | --- | --- | --- | --- | --- | --- | --- |
|  | | B | SE | Wald | free degree | conspicuousness |  |  |  |
|  |  |  |  |  |  |  |  |  |  |
| Step 1 | A IPH | 2.123 | .388 | 29.993 | 1 | .000 |  |  |  |

| **Variables not included in Eq^a^** | | | | |
| --- | --- | --- | --- | --- |
|  | | score | free degree | conspicuousness |
| Step 1 | A age | .156 | 1 | .692 |
|  | A Gender: 0= female; 1= male | .558 | 1 | .455 |
|  | Pre-addmision mRS | 1.575 | 1 | .210 |
|  | A7. Baseline NIHSS | .548 | 1 | .459 |

| a.Residual chi-square = 2.433, degree of freedom of 4, significance =.657 |
| --- |

| **Model (if the item is removed)** | | | | |
| --- | --- | --- | --- | --- |
| Item has been removed | | Chi square loss | free degree | conspicuousness |
| Step 1 | A IPH | 29.280 | 1 | .000 |

| **Mean values of covariates** | |
| --- | --- |
|  | average value |
| A age | 60.189 |
| A Gender: 0= female; 1= male | .308 |
| Pre-addmision mRS | 1.408 |
| A7. Baseline NIHSS | 4.793 |
| A IPH | .178 |

COXREG survevalmonth

/STATUS=outcome2('1') /CONTRAST(strokeorTIA)=Indicator /CONTRAST(smoke)=Indicator

/CONTRAST(CAD_01)=Indicator /CONTRAST(DM_01)=Indicator /CONTRAST(familyhistory_stroke)=Indicator

/ CONTRAST (gender) =Indicator / CONTRAST (hyperlipidemia) =Indicator / CONTRAST (HBP_01) =Indicator

/ METHOD=FSTEP (LR) age_01 gender preaddmisionmRS_01 NIHSS_A IPH1 HBP_01 DM_01 CAD_01 Hyperlipidaemia strokeorTIA

familyhistory_stroke smoke

/PRINT=CI(95)

/CRITERIA=PIN(.05) POUT(.10) ITERATE(20).

Cox regression

| **remarks** | | |
| --- | --- | --- |
| The output has been created | | 22-MAY-2022 11:16:53 |
| explanatory note | |  |
| import | data | D: \ Che Fengli is important!!!\ CCA raw data \ AA-CCA-IPH related factors \ Aa Che-survival status.sav-2022-2-25.sav |
|  | Activity dataset | data set 1 |
|  | filter | <not have> |
|  | weight | <not have> |
|  | Split files | <not have> |
|  | Number of rows in the working data file | 171 |
| Missing value processing | Definition of deletions | Treat the user-defined missing values as missing. |
| grammar | | COXREG survevalmonth  /STATUS=outcome2('1') /CONTRAST(strokeorTIA)=Indicator /CONTRAST(smoke)=Indicator  /CONTRAST(CAD_01)=Indicator /CONTRAST(DM_01)=Indicator /CONTRAST(familyhistory_stroke)=Indicator  / CONTRAST (gender) =Indicator / CONTRAST (hyperlipidemia) =Indicator / CONTRAST (HBP_01) =Indicator  / METHOD=FSTEP (LR) age_01 gender preaddmisionmRS_01 NIHSS_A IPH1 HBP_01 DM_01 CAD_01 Hyperlipidaemia strokeorTIA  familyhistory_stroke smoke  /PRINT=CI(95)  /CRITERIA=PIN(.05) POUT(.10) ITERATE(20). |
| resource | Handler time | 00:00:00.02 |
|  | It takes time | 00:00:00.01 |

| **Case handling summary** | | | |
| --- | --- | --- | --- |
|  | | The number of cases | percentage |
| Cases that can be used in the analysis | event^a^ | 28 | 16.4% |
|  | After checking out | 141 | 82.5% |
|  | amount to | 169 | 98.8% |
| Deleted cases | Cases with missing values | 2 | 1.2% |
|  | Cases with a negative time period | 0 | 0.0% |
|  | The first incident in the layer | 0 | 0.0% |
|  | amount to | 2 | 1.2% |
| amount to | | 171 | 100.0% |

| a.Dependent variable: survevalmonth |
| --- |

| **Classification variable coding^a,c,d,e,f,g,h,i^** | | | |
| --- | --- | --- | --- |
|  | | frequency | (1) |
| A Gender: 0= female; 1= male^b^ | 0=female | 52 | 1 |
|  | 1=male | 117 | 0 |
| A Hypertension: 1. Yes 0. No 9. Unknown^b^ | 0=no | 54 | 1 |
|  | 1=yes | 115 | 0 |
| A DM 1. Yes 0. No 9. Unknown^b^ | 0 | 119 | 1 |
|  | 1 | 50 | 0 |
| A CVD: 1. Yes 0. No 9^b^ | 0 | 150 | 1 |
|  | 1 | 19 | 0 |
| A Hyperlipidemia: 1. Yes 0. No 9. Unknown^b^ | 0= 0 | 113 | 1 |
|  | 1= 1 | 56 | 0 |
| A Family history^b^ | 0= No | 145 | 1 |
|  | 1= Yes | 24 | 0 |
| A History of previous stroke: 1. 0. No 9^b^ | 0 | 142 | 1 |
|  | 1 | 27 | 0 |
| 9. Current smoking^b^ | 0= Never smoking | 83 | 1 |
|  | 1= Is | 86 | 0 |

| a.Category variable: ^ 1 (gender) |
| --- |
| b.Indicator parameter encoding |
| c.Category variable: ^ 1, (HBP_01) |
| d.Category variable: ^ 1 (DM_01) |
| e.Category variable: ^ 1, (CAD_01) |
| f.Category variable: ^ 1 (hyperlipidemia) |
| g.Category variable: ^ 1 (familyhistory_stroke) |
| h.Category variable: ^ 1 (strokeorTIA) |
| i.Category variable: ^ 1 (smoke) |

Block 0: Start block

| **Variables not included in Eq^a^** | | | |
| --- | --- | --- | --- |
|  | score | free degree | conspicuousness |
| A age | 2.045 | 1 | .153 |
| A Gender: 0= female; 1= male | 1.316 | 1 | .251 |
| Pre-addmision mRS | 4.726 | 1 | .030 |
| A7. Baseline NIHSS | 4.188 | 1 | .041 |
| A IPH | 42.959 | 1 | .000 |
| A Hypertension: 1. Yes 0. No 9. Unknown | .271 | 1 | .602 |
| A DM 1. Yes 0. No 9. Unknown | .496 | 1 | .481 |
| A CVD: 1. Yes 0. No 9 | 1.757 | 1 | .185 |
| A Hyperlipidemia: 1. Yes 0. No 9. Unknown | .218 | 1 | .641 |
| A History of previous stroke: 1. 0. No 9 | .537 | 1 | .464 |
| A Family history | 11.099 | 1 | .001 |
| 9. Current smoking | 9.474 | 1 | .002 |

| a.Residual chi-square = 58.206, degree of freedom of 12, significance =.000 |
| --- |

Block 1: Method = Step forward (likelihood ratio)

| **The Omnibus's test of the model coefficients^c^** | | | | | | | | | | |
| --- | --- | --- | --- | --- | --- | --- | --- | --- | --- | --- |
| step | -2 log-likelihood | Overall (score) | | | Make the changes from before | | |  |  |  |
|  |  | chi-square | free degree | conspicuousness | chi-square | free degree | conspicuousness |  |  |  |
| 1^a^ | 245.693 | 42.959 | 1 | .000 | 29.280 | 1 | .000 |  |  |  |
| 2^b^ | 241.250 | 49.636 | 2 | .000 | 4.443 | 1 | .035 |  |  |  |

| **Variables in Eq** | | | | | | | | | |
| --- | --- | --- | --- | --- | --- | --- | --- | --- | --- |
|  | | B | SE | Wald | free degree | conspicuousness |  |  |  |
|  |  |  |  |  |  |  |  |  |  |
| Step 1 | A IPH | 2.123 | .388 | 29.993 | 1 | .000 |  |  |  |
| Step 2 | A IPH | 1.995 | .394 | 25.672 | 1 | .000 |  |  |  |
|  | A Family history | -.890 | .401 | 4.917 | 1 | .027 |  |  |  |

| **Variables not included in Eq^a,b^** | | | | |
| --- | --- | --- | --- | --- |
|  | | score | free degree | conspicuousness |
| Step 1 | A age | .156 | 1 | .692 |
|  | A Gender: 0= female; 1= male | .558 | 1 | .455 |
|  | Pre-addmision mRS | 1.575 | 1 | .210 |
|  | A7. Baseline NIHSS | .548 | 1 | .459 |
|  | A Hypertension: 1. Yes 0. No 9. Unknown | .948 | 1 | .330 |
|  | A DM 1. Yes 0. No 9. Unknown | .111 | 1 | .739 |
|  | A CVD: 1. Yes 0. No 9 | 1.672 | 1 | .196 |
|  | A Hyperlipidemia: 1. Yes 0. No 9. Unknown | .002 | 1 | .967 |
|  | A History of previous stroke: 1. 0. No 9 | 1.104 | 1 | .293 |
|  | A Family history | 5.229 | 1 | .022 |
|  | 9. Current smoking | 4.583 | 1 | .032 |
| Step 2 | A age | 1.184 | 1 | .277 |
|  | A Gender: 0= female; 1= male | 1.081 | 1 | .299 |
|  | Pre-addmision mRS | 1.244 | 1 | .265 |
|  | A7. Baseline NIHSS | .454 | 1 | .500 |
|  | A Hypertension: 1. Yes 0. No 9. Unknown | .211 | 1 | .646 |
|  | A DM 1. Yes 0. No 9. Unknown | .231 | 1 | .631 |
|  | A CVD: 1. Yes 0. No 9 | 1.119 | 1 | .290 |
|  | A Hyperlipidemia: 1. Yes 0. No 9. Unknown | .008 | 1 | .928 |
|  | A History of previous stroke: 1. 0. No 9 | .971 | 1 | .325 |
|  | 9. Current smoking | 2.926 | 1 | .087 |

| a.Residual chi-square = 13.547, degree of freedom of 11, significance =.259 |
| --- |
| b.Residual chi-square = 9.068, degree of freedom of 10, significance =.526 |

| **Model (if the item is removed)** | | | | |
| --- | --- | --- | --- | --- |
| Item has been removed | | Chi square loss | free degree | conspicuousness |
| Step 1 | A IPH | 29.280 | 1 | .000 |
| Step 2 | A IPH | 25.386 | 1 | .000 |
|  | A Family history | 4.443 | 1 | .035 |

| **Mean values of covariates** | |
| --- | --- |
|  | average value |
| A age | 60.189 |
| A Gender: 0= female; 1= male | .308 |
| Pre-addmision mRS | 1.408 |
| A7. Baseline NIHSS | 4.793 |
| A IPH | .178 |
| A Hypertension: 1. Yes 0. No 9. Unknown | .320 |
| A DM 1. Yes 0. No 9. Unknown | .704 |
| A CVD: 1. Yes 0. No 9 | .888 |
| A Hyperlipidemia: 1. Yes 0. No 9. Unknown | .669 |
| A History of previous stroke: 1. 0. No 9 | .840 |
| A Family history | .858 |
| 9. Current smoking | .491 |

COXREG survevalmonth

/STATUS=outcome2('1') /CONTRAST(CAD_01)=Indicator /CONTRAST(statinbeforeoutcome)=Indicator

/ CONTRAST (DM_01) =Indicator / CONTRAST (familyhistory_stroke) =Indicator / CONTRAST (hyperlipidemia) =Indicator

/CONTRAST(strokeorTIA)=Indicator /CONTRAST(smoke)=Indicator

/CONTRAST(asprinbeforeoutcome)=Indicator /CONTRAST(gender)=Indicator /CONTRAST(HBP_01)=Indicator

/ METHOD=FSTEP (LR) age_01 gender preaddmisionmRS_01 NIHSS_A IPH1 HBP_01 DM_01 CAD_01 Hyperlipidaemia strokeorTIA

familyhistory_stroke smoke stenosisrate_area1 asprinbeforeoutcome statinbeforeoutcome

Calcification_binary01 LooseMatrix_binary_00 LRNC_binary01 FCR

/PRINT=CI(95)

/CRITERIA=PIN(.05) POUT(.10) ITERATE(20).

Cox regression

| **remarks** | | |
| --- | --- | --- |
| The output has been created | | 22-MAY-2022 11:19:00 |
| explanatory note | |  |
| import | data | D: \ Che Fengli is important!!!\ CCA raw data \ AA-CCA-IPH related factors \ Aa Che-survival status.sav-2022-2-25.sav |
|  | Activity dataset | data set 1 |
|  | filter | <not have> |
|  | weight | <not have> |
|  | Split files | <not have> |
|  | Number of rows in the working data file | 171 |
| Missing value processing | Definition of deletions | Treat the user-defined missing values as missing. |
| grammar | | COXREG survevalmonth  /STATUS=outcome2('1') /CONTRAST(CAD_01)=Indicator /CONTRAST(statinbeforeoutcome)=Indicator  / CONTRAST (DM_01) =Indicator / CONTRAST (familyhistory_stroke) =Indicator / CONTRAST (hyperlipidemia) =Indicator  /CONTRAST(strokeorTIA)=Indicator /CONTRAST(smoke)=Indicator  /CONTRAST(asprinbeforeoutcome)=Indicator /CONTRAST(gender)=Indicator /CONTRAST(HBP_01)=Indicator  / METHOD=FSTEP (LR) age_01 gender preaddmisionmRS_01 NIHSS_A IPH1 HBP_01 DM_01 CAD_01 Hyperlipidaemia strokeorTIA  familyhistory_stroke smoke stenosisrate_area1 asprinbeforeoutcome statinbeforeoutcome  Calcification_binary01 LooseMatrix_binary_00 LRNC_binary01 FCR  /PRINT=CI(95)  /CRITERIA=PIN(.05) POUT(.10) ITERATE(20). |
| resource | Handler time | 00:00:00.02 |
|  | It takes time | 00:00:00.02 |

| **Case handling summary** | | | |
| --- | --- | --- | --- |
|  | | The number of cases | percentage |
| Cases that can be used in the analysis | event^a^ | 28 | 16.4% |
|  | After checking out | 141 | 82.5% |
|  | amount to | 169 | 98.8% |
| Deleted cases | Cases with missing values | 2 | 1.2% |
|  | Cases with a negative time period | 0 | 0.0% |
|  | The first incident in the layer | 0 | 0.0% |
|  | amount to | 2 | 1.2% |
| amount to | | 171 | 100.0% |

| a.Dependent variable: survevalmonth |
| --- |

| **Classification variable coding^a,c,d,e,f,g,h,i,j,k^** | | | |
| --- | --- | --- | --- |
|  | | frequency | (1) |
| A Gender: 0= female; 1= male^b^ | 0=female | 52 | 1 |
|  | 1=male | 117 | 0 |
| A Hypertension: 1. Yes 0. No 9. Unknown^b^ | 0=no | 54 | 1 |
|  | 1=yes | 115 | 0 |
| A DM 1. Yes 0. No 9. Unknown^b^ | 0 | 119 | 1 |
|  | 1 | 50 | 0 |
| A CVD: 1. Yes 0. No 9^b^ | 0 | 150 | 1 |
|  | 1 | 19 | 0 |
| A Hyperlipidemia: 1. Yes 0. No 9. Unknown^b^ | 0= 0 | 113 | 1 |
|  | 1= 1 | 56 | 0 |
| A Family history^b^ | 0= No | 145 | 1 |
|  | 1= Have | 24 | 0 |
| A History of previous stroke: 1. 0. No 9^b^ | 0 | 142 | 1 |
|  | 1 | 27 | 0 |
| 9. Current smoking^b^ | 0= Never smoking | 83 | 1 |
|  | 1= Is | 86 | 0 |
| Taking aspirin until primary outcome: 0= no; 1= yes; 9. Unknown^b^ | 0= No | 75 | 1 |
|  | 1= Have | 94 | 0 |
| Taking statins until primary outcome: 0= no; 1= yes; 9. Unknown^b^ | 0 | 99 | 1 |
|  | 1 | 70 | 0 |

| a.Category variable: ^ 1 (gender) |
| --- |
| b.Indicator parameter encoding |
| c.Category variable: ^ 1, (HBP_01) |
| d.Category variable: ^ 1 (DM_01) |
| e.Category variable: ^ 1, (CAD_01) |
| f.Category variable: ^ 1 (hyperlipidemia) |
| g.Category variable: ^ 1 (familyhistory_stroke) |
| h.Category variable: ^ 1 (strokeorTIA) |
| i.Category variable: ^ 1 (smoke) |
| j.Category variable: ^ 1 (asprinbeforeoutcome) |
| k.Category variable: ^ 1 (statinbeforeoutcome) |

Block 0: Start block

| **Variables not included in Eq^a^** | | | |
| --- | --- | --- | --- |
|  | score | free degree | conspicuousness |
| A age | 2.045 | 1 | .153 |
| A Gender: 0= female; 1= male | 1.316 | 1 | .251 |
| Pre-addmision mRS | 4.726 | 1 | .030 |
| A7. Baseline NIHSS | 4.188 | 1 | .041 |
| A IPH | 42.959 | 1 | .000 |
| A Hypertension: 1. Yes 0. No 9. Unknown | .271 | 1 | .602 |
| A DM 1. Yes 0. No 9. Unknown | .496 | 1 | .481 |
| A CVD: 1. Yes 0. No 9 | 1.757 | 1 | .185 |
| A Hyperlipidemia: 1. Yes 0. No 9. Unknown | .218 | 1 | .641 |
| A History of previous stroke: 1. Yes 0. No 9 | .537 | 1 | .464 |
| A Family history | 11.099 | 1 | .001 |
| 9. Current smoking | 9.474 | 1 | .002 |
| Stenosis degree (area calculation method 1): LAmin / LAmin + WAmin | .659 | 1 | .417 |
| Taking aspirin until primary outcome: 0= no; 1= yes; 9. Unknown | 1.910 | 1 | .167 |
| Taking statins until primary outcome: 0= no; 1= yes; 9. Unknown | 5.944 | 1 | .015 |
| Acalcification | .642 | 1 | .423 |
| A loose matrix | 10.070 | 1 | .002 |
| A LRNC | 3.039 | 1 | .081 |
| A FCR: 0= No;1= Yes | .964 | 1 | .326 |

| a.Residual chi-square = 72.303, degree of freedom of 19, significance =.000 |
| --- |

Block 1: Method = Step forward (likelihood ratio)

| **The Omnibus's test of the model coefficients^e^** | | | | | | | | | | |
| --- | --- | --- | --- | --- | --- | --- | --- | --- | --- | --- |
| step | -2 log-likelihood | Overall (score) | | | Make the changes from before | | |  |  |  |
|  |  | chi-square | free degree | conspicuousness | chi-square | free degree | conspicuousness |  |  |  |
| 1^a^ | 245.693 | 42.959 | 1 | .000 | 29.280 | 1 | .000 |  |  |  |
| 2^b^ | 241.250 | 49.636 | 2 | .000 | 4.443 | 1 | .035 |  |  |  |
| 3^c^ | 234.541 | 54.539 | 3 | .000 | 6.709 | 1 | .010 |  |  |  |
| 4^d^ | 227.227 | 59.187 | 4 | .000 | 7.314 | 1 | .007 |  |  |  |

| **Variables in Eq** | | | | | | | | | |
| --- | --- | --- | --- | --- | --- | --- | --- | --- | --- |
|  | | B | SE | Wald | free degree | conspicuousness |  |  |  |
|  |  |  |  |  |  |  |  |  |  |
| Step 1 | A IPH | 2.123 | .388 | 29.993 | 1 | .000 |  |  |  |
| Step 2 | A IPH | 1.995 | .394 | 25.672 | 1 | .000 |  |  |  |
|  | A Family history | -.890 | .401 | 4.917 | 1 | .027 |  |  |  |
| Step 3 | A IPH | 1.849 | .393 | 22.173 | 1 | .000 |  |  |  |
|  | A Family history | -1.022 | .399 | 6.547 | 1 | .011 |  |  |  |
|  | A loose matrix | 1.072 | .442 | 5.876 | 1 | .015 |  |  |  |
| Step 4 | A IPH | 2.090 | .406 | 26.523 | 1 | .000 |  |  |  |
|  | A Family history | -1.163 | .409 | 8.092 | 1 | .004 |  |  |  |
|  | Taking aspirin until primary outcome: 0= no; 1= yes; 9. Unknown | 1.115 | .414 | 7.240 | 1 | .007 |  |  |  |
|  | A loose matrix | 1.282 | .459 | 7.797 | 1 | .005 |  |  |  |

| **Variables not included in Eq^a,b,c,d^** | | | | |
| --- | --- | --- | --- | --- |
|  | | score | free degree | conspicuousness |
| Step 1 | A age | .156 | 1 | .692 |
|  | A Gender: 0= female; 1= male | .558 | 1 | .455 |
|  | Pre-addmision mRS | 1.575 | 1 | .210 |
|  | A7. Baseline NIHSS | .548 | 1 | .459 |
|  | A Hypertension: 1. Yes 0. No 9. Unknown | .948 | 1 | .330 |
|  | A DM 1. Yes 0. No 9. Unknown | .111 | 1 | .739 |
|  | A CVD: 1. Yes 0. No 9 | 1.672 | 1 | .196 |
|  | A Hyperlipidemia: 1. Yes 0. No 9. Unknown | .002 | 1 | .967 |
|  | A History of previous stroke: 1. 0. No 9 | 1.104 | 1 | .293 |
|  | A Family history | 5.229 | 1 | .022 |
|  | 9. Current smoking | 4.583 | 1 | .032 |
|  | Stenosis degree (area calculation method 1): LAmin / LAmin + WAmin | 1.316 | 1 | .251 |
|  | Taking aspirin until primary outcome: 0= no; 1= yes; 9. Unknown | 5.060 | 1 | .024 |
|  | Taking statins until primary outcome: 0= no; 1= yes; 9. Unknown | 3.025 | 1 | .082 |
|  | Acalcification | 1.385 | 1 | .239 |
|  | A loose matrix | 5.081 | 1 | .024 |
|  | A LRNC | .215 | 1 | .643 |
|  | A FCR: 0= No;1= Yes | .455 | 1 | .500 |
| Step 2 | A age | 1.184 | 1 | .277 |
|  | A Gender: 0= female; 1= male | 1.081 | 1 | .299 |
|  | Pre-addmision mRS | 1.244 | 1 | .265 |
|  | A7. Baseline NIHSS | .454 | 1 | .500 |
|  | A Hypertension: 1. Yes 0. No 9. Unknown | .211 | 1 | .646 |
|  | A DM 1. Yes 0. No 9. Unknown | .231 | 1 | .631 |
|  | A CVD: 1. Yes 0. No 9 | 1.119 | 1 | .290 |
|  | A Hyperlipidemia: 1. Yes 0. No 9. Unknown | .008 | 1 | .928 |
|  | A History of previous stroke: 1. 0. No 9 | .971 | 1 | .325 |
|  | 9. Current smoking | 2.926 | 1 | .087 |
|  | Stenosis degree (area calculation method 1): LAmin / LAmin + WAmin | .582 | 1 | .445 |
|  | Taking aspirin until primary outcome: 0= no; 1= yes; 9. Unknown | 5.296 | 1 | .021 |
|  | Taking statins until primary outcome: 0= no; 1= yes; 9. Unknown | 2.463 | 1 | .117 |
|  | Acalcification | .980 | 1 | .322 |
|  | A loose matrix | 6.412 | 1 | .011 |
|  | A LRNC | .209 | 1 | .648 |
|  | A FCR: 0= No;1= Yes | .486 | 1 | .486 |
| Step 3 | A age | .580 | 1 | .446 |
|  | A Gender: 0= female; 1= male | .570 | 1 | .450 |
|  | Pre-addmision mRS | 2.392 | 1 | .122 |
|  | A7. Baseline NIHSS | .744 | 1 | .388 |
|  | A Hypertension: 1. Yes 0. No 9. Unknown | .071 | 1 | .790 |
|  | A DM 1. Yes 0. No 9. Unknown | .057 | 1 | .812 |
|  | A CVD: 1. Yes 0. No 9 | .649 | 1 | .420 |
|  | A Hyperlipidemia: 1. Yes 0. No 9. Unknown | .038 | 1 | .845 |
|  | A History of previous stroke: 1. 0. No 9 | 1.162 | 1 | .281 |
|  | 9. Current smoking | 2.634 | 1 | .105 |
|  | Stenosis degree (area calculation method 1): LAmin / LAmin + WAmin | .987 | 1 | .321 |
|  | Taking aspirin until primary outcome: 0= no; 1= yes; 9. Unknown | 7.824 | 1 | .005 |
|  | Taking statins until primary outcome: 0= no; 1= yes; 9. Unknown | 2.459 | 1 | .117 |
|  | Acalcification | 2.033 | 1 | .154 |
|  | A LRNC | .023 | 1 | .880 |
|  | A FCR: 0= No;1= Yes | .464 | 1 | .496 |
| Step 4 | A age | .000 | 1 | .993 |
|  | A Gender: 0= female; 1= male | .862 | 1 | .353 |
|  | Pre-addmision mRS | 1.511 | 1 | .219 |
|  | A7. Baseline NIHSS | .614 | 1 | .433 |
|  | A Hypertension: 1. Yes 0. No 9. Unknown | .016 | 1 | .900 |
|  | A DM 1. Yes 0. No 9. Unknown | .492 | 1 | .483 |
|  | A CVD: 1. Yes 0. No 9 | .306 | 1 | .580 |
|  | A Hyperlipidemia: 1. Yes 0. No 9. Unknown | .368 | 1 | .544 |
|  | A History of previous stroke: 1. 0. No 9 | 1.136 | 1 | .286 |
|  | 9. Current smoking | 2.100 | 1 | .147 |
|  | Stenosis degree (area calculation method 1): LAmin / LAmin + WAmin | 1.902 | 1 | .168 |
|  | Taking statins until primary outcome: 0= no; 1= yes; 9. Unknown | .454 | 1 | .500 |
|  | Acalcification | 1.751 | 1 | .186 |
|  | A LRNC | .069 | 1 | .793 |
|  | A FCR: 0= No;1= Yes | .608 | 1 | .435 |

| a.Residual chi-square = 32.568, degree of freedom of 18, significance =.019 |
| --- |
| b.Residual chi-square = 28.364, degree of freedom of 17, significance =.041 |
| c.Residual chi-square = 20.694, degree of freedom of 16, significance =.191 |
| d.Residual chi-square = 12.286, degree of freedom of 15, significance =.657 |

| **Model (if the item is removed)** | | | | |
| --- | --- | --- | --- | --- |
| Item has been removed | | Chi square loss | free degree | conspicuousness |
| Step 1 | A IPH | 29.280 | 1 | .000 |
| Step 2 | A IPH | 25.386 | 1 | .000 |
|  | A Family history | 4.443 | 1 | .035 |
| Step 3 | A IPH | 22.210 | 1 | .000 |
|  | A Family history | 5.785 | 1 | .016 |
|  | A loose matrix | 6.709 | 1 | .010 |
| Step 4 | A IPH | 26.535 | 1 | .000 |
|  | A Family history | 7.106 | 1 | .008 |
|  | Taking aspirin until primary outcome: 0= no; 1= yes; 9. Unknown | 7.314 | 1 | .007 |
|  | A loose matrix | 9.046 | 1 | .003 |

| **Mean values of covariates** | |
| --- | --- |
|  | average value |
| A age | 60.189 |
| A Gender: 0= female; 1= male | .308 |
| Pre-addmision mRS | 1.408 |
| A7. Baseline NIHSS | 4.793 |
| A IPH | .178 |
| A Hypertension: 1. Yes 0. No 9. Unknown | .320 |
| A DM 1. Yes 0. No 9. Unknown | .704 |
| A CVD: 1. Yes 0. No 9 | .888 |
| A Hyperlipidemia: 1. Yes 0. No 9. Unknown | .669 |
| A History of previous stroke: 1. 0. No 9 | .840 |
| A Family history | .858 |
| 9. Current smoking | .491 |
| Stenosis degree (area calculation method 1): LAmin / LAmin + WAmin | .340 |
| Taking aspirin until primary outcome: 0= no; 1= yes; 9. Unknown | .444 |
| Taking statins until primary outcome: 0= no; 1= yes; 9. Unknown | .586 |
| Acalcification | .544 |
| A loose matrix | .467 |
| A LRNC | .811 |
| A FCR: 0= No;1= Yes | .036 |

DISPLAY DICTIONARY.

**File Information**

| **Notes** | | |
| --- | --- | --- |
| Output Created | | 15-JUN-2022 18:24:08 |
| Comments | |  |
| Input | Active Dataset | DataSet0 |
|  | Filter | <none> |
|  | Weight | <none> |
|  | Split File | <none> |
| Syntax | | DISPLAY DICTIONARY. |
| Resources | Processor Time | 00:00:00.00 |
|  | Elapsed Time | 00:00:00.00 |

[DataSet0]

| **Warnings** |
| --- |
| No variables are defined.The DISPLAY command cannot be executed except for displaying information about macros. |
