# Supplementary material for: Extracranial carotid plaque hemorrhage predicts ipsilateral stroke recurrence in patients with carotid atherosclerosis – a study based on high-resolution vessel wall imaging MRI
Source: BMC Neurol. 2022 Jun 28;22:237. doi: 10.1186/s12883-022-02758-3 (PMC9238155; doi:10.1186/s12883-022-02758-3)
Supplement: Supplementary file 1 — Additional file 1. [file 12883_2022_2758_MOESM1_ESM.zip › Supplementary/C-supllementary1-Non-standard Abbreviations and Acronyms.docx]

**Non-standard Abbreviations and Acronyms**

| IPH | intraplaque hemorrhage |
| --- | --- |
| CNSR | China National Stroke Registry |
| CICAS | China Intracranial Atherosclerosis study |
| MRI | magnetic resonance imaging |
| HR VWMRI | high-resolution vessel wall MRI |
| PET | positron emission tomography |
| CT | computed tomography |
| IMT | intima-media-thickness |
| NIHSS | national institutes of health stroke scale |
| mRS | modified Rankin scale |
| MRA | magnetic resonance angiography |
| 3D-TOF | three-dimensional time of flight |
| T1W | T1-weighted |
| T2W | T2-weighted |
| MPRAGE | magnetization-prepared rapid acquisition gradient echo |
| GD-DTPA | gadolinium diethylenetriamine pentametric acid |
| SPAIR | spectral preservation attenuated inversion recovery |
| TR | repetition time |
| TE | echo time |
| FA | flip angle |
| FOV | field of view |
| SNR | signal-noise ratio |
| DWI | diffusion-weighted images |
| SDs | standard deviations |
| IQRs | interquartile ranges |
| ICC | intraclass correlation coefficient |
| ROC | receiver–operator characteristic curve |
| HR | hazard ratio |
| OR | odds ratio |
| CI | confidence interval |
| AUC | area-under-the-curve |
| HBP | hypertension |
| DM | diabetes mellitus |
| CHD | coronary heart disease |
| SBP | systolic blood pressure |
| DBP | diastolic blood pressure |
| hs-CRP | hypersensitive c-reactive protein |
| TC | cholesterol |
| TG | triglycerides |
| LDL-C | low-density lipoprotein cholesterol |
| HDL-C | high-density lipoprotein cholesterol |
| Hcy | homocysteine |
| ICC | intraclass correlation coefficient |
| CA | carotid artery |
| LA | lumen area |
| WA | wall area |
| VA | vessel area |
| NWI | normalized wall index |
| WT | wall thickness |
| PA | plaque area |
| RI | remodeling index |
| LRNC | lipid-rich necrotic core |
| FCR | fibrous cap rupture |
